# Low‐frequency stimulation of group III and IV hind limb afferents evokes reflex pressor responses in decerebrate rats

**DOI:** 10.14814/phy2.13001

**Published:** 2016-10-24

**Authors:** Jonathan E. Harms, Steven W. Copp, Marc P. Kaufman

**Affiliations:** ^1^ Heart and Vascular InstitutePenn State College of MedicineHersheyPennsylvania

**Keywords:** Afferent nerve, exercise pressor reflex, femoral artery occlusion, peripheral artery disease

## Abstract

Contraction of freely perfused hind limb muscles in decerebrate rats evokes the exercise pressor reflex, resulting in sympathetic activation and increased blood pressure. This reflex is propagated along mechanically sensitive group III and metabolically sensitive group IV afferent nerve fibers. Recent research by our laboratory has focused on the exaggeration of the exercise pressor reflex in decerebrate rats with simulated peripheral artery disease, which was induced by ligating the femoral artery for 72 h before the start of the experiment. Recently, we showed that ligating the femoral artery increased the responses of single fiber group III and IV triceps surae muscle afferents to static contraction. The objective of this study was to determine if electrical stimulation of group III and IV afferents at frequencies approximating those occurring during static contraction was capable of reflexively increasing arterial blood pressure. We directly stimulated muscle afferents in the absence of muscle contraction for both freely perfused and ligated rats. We established 0.25 Hz as the minimal stimulation frequency to observe a sustained blood pressure response. The blood pressure response increased in a graded fashion as both stimulus frequency and motor threshold were increased. Additionally, we observed similar blood pressure responses from both freely perfused and ligated rats, suggesting that spinal and medullary processing of group III and IV afferent input plays no role in augmenting the pressor response to contraction caused by femoral artery ligation.

## Introduction

A reflex arising from contracting hind limb skeletal muscles is an important neural mechanism that contributes to the cardiovascular adjustments to exercise (Coote et al. 1971; McCloskey and Mitchell 1972). These adjustments, which include increases in peripheral vascular resistance and cardiac contractility and rate, function to increase arterial blood flow and oxygen to the exercising muscles. This increase, in turn, supports the ability of the muscle to contract. This neural mechanism has been named the exercise pressor reflex (Mitchell et al. 1983) and its afferent arm is comprised of group III and IV fibers whose endings are located in and near the muscle interstitium (Coote and Perez‐Gonzalez 1970; McCloskey and Mitchell 1972).

In patients with peripheral artery disease (PAD) the exercise pressor reflex is exaggerated (Baccelli et al. 1999; Muller et al. 2012). Terjung and colleagues (Yang et al. 1990; Prior et al. 2004; Taylor et al. 2008) have developed an animal model that simulates the arterial blood flow patterns seen in peripheral artery disease in humans. In this model, the femoral artery of one hind limb is ligated for 72 h. At rest, arterial blood flow to the hind limb whose femoral artery is ligated is adequate because its collateral circulation is sufficient to meet its low metabolic demand. During exercise, however, arterial blood flow is inadequate because its collateral circulation cannot meet the increased metabolic demand of the working muscles, resulting in ischemia.

In decerebrate unanesthetized rats, static contraction of the hind limb muscles whose femoral arteries were ligated for 72 h evoked a significantly larger exercise pressor reflex than did static contraction of the contralateral hind limb muscles whose femoral arteries were freely perfused (Tsuchimochi et al. 2010). In addition, static contraction of the gastrocnemius muscles in rats with ligated femoral arteries evoked significantly greater responses from group III and IV muscle afferents, which comprise the afferent arm of the reflex, than did static contraction of these muscles in rats whose femoral arteries were freely perfused (Stone et al. 2015). Although significant, these responses were not large, averaging about 0.5 impulses per second in rats with freely perfused femoral arteries and 1.0 impulses per second in rats with ligated femoral arteries (Stone et al. 2015). Consequently, these findings raised the question as to whether these relatively small increases in thin fiber afferent activity were sufficient to evoke reflex increases in arterial blood pressure. In addition, the question remained as to whether ligation of a femoral artery for 72 h had an effect on central neural processing of primary afferent input arising from exercising muscles. These questions prompted us to determine the effect of electrical stimulation of group III and IV afferent fibers on arterial blood pressure in decerebrate paralyzed rats.

## Materials and Methods

All procedures were approved by the Institutional Care and Use Committee of the Pennsylvania State University College of Medicine. Experiments were conducted on 54 male, Sprague–Dawley rats, ranging from 12 to 14 weeks in age and weighing 350–450 g. Seventy‐two hours before the experiment, a group of rats underwent surgery to induce unilateral femoral artery ligation. The rats were anesthetized with a mixture of 4% isoflurane and 100% oxygen. The femoral artery was isolated and 5–0 suture was tied tightly around it approximately 3 mm distal to the inguinal ligament. Rats were allowed 72 h to recover following the femoral artery occlusion procedure, which does not affect normal cage activity (Taylor et al. 2008). Rats that underwent this surgery will be referred to as “ligated” whereas those who did not have their femoral artery ligated will be referred to as “freely perfused”.

On the day of the experiment, rats were anesthetized with isoflurane (4%) in oxygen. The trachea was cannulated and the lungs mechanically ventilated with the gaseous anesthetic (2% isoflurane). The carotid arteries and one jugular vein were cannulated (PE‐50) to measure arterial blood pressure and to administer drugs and fluids, respectively. Arterial blood gases and pH were measured using an automated blood gas analyzer (ABL 80, Radiometer, Brea, CA, USA). ρCO_2_ and arterial pH were maintained within normal ranges either by adjusting ventilation and oxygen or by an intravenous injection of sodium bicarbonate (8.5%). Body temperature was maintained between 36.5 and 38.0°C by a heat lamp. Arterial blood pressure was measured by attaching one carotid cannula to a Statham P23XL strain gauge (AMETEK Power Instruments, Rochester, NY, USA). Heart rate was calculated from the arterial pressure pulse (Spike 2; Cambridge Electronics Design, Cambridge, UK).

The rats were secured in a Kopf customized spinal frame by clamps placed on the rostral lumbar vertebra, the pelvis, and the left ankle. A precollicular decerebration was performed and all neural tissue rostral to the superior colliculi was removed (Smith et al. 2001; Tsuchimochi et al. 2010). Bleeding was controlled and the cranial vault was filled with gauze. Immediately after the decerebration, anesthesia was discontinued. The tibial nerve was isolated, a shielded stimulating electrode was placed underneath it, and the leg was covered in saline saturated gauze.

In the first protocol, we determined the minimum frequency needed to evoke a reflexive increase in arterial blood pressure when stimulating hind limb afferents. To accomplish this, we placed the stimulating electrode on the tibial nerve just as it exited the triceps surae muscles. We electrically stimulated (Grass S88) the tibial nerve for 30 sec with the following frequencies (Hz): 0.1, 0.25, 0.5, 1.0, 3.0, and 5.0 applied in random order. The pulse duration was always 0.01 msec. The current intensity used was randomly selected from 5, 20, or 100 times the current needed to evoke a muscle twitch when a single pulse was applied to the tibial nerve. Only one of the specified current intensities was used for each rat, which was either freely perfused or ligated. In total, 24 freely perfused and an additional 24 ligated rats (eight per current intensity) were used in this first protocol. In these experiments, the rats were paralyzed with pancuronium bromide (0.5 mg/kg). Baseline blood pressure and heart rate values were calculated from the maximum of the 30 sec‐period prior to stimulation onset. Peak change was calculated as the baseline subtracted maximum value during stimulation.

In the second protocol, we measured the compound action potential recorded from filaments split from the L4 or L5 dorsal roots in order to calculate the conduction velocities of the hind limb afferents activated by stimulating the tibial nerve with pulses (0.01 msec) that were 5, 20, and 100 times the motor threshold. To accomplish this task, a lumbar and sacral laminectomy was performed and the L4 and L5 dorsal roots, which provide a sensory innervation to the hind limb, were isolated. The rats (three freely perfused and three ligated) were paralyzed with pancuronium bromide (0.5 mg/kg). The evoked compound action potential was passed through a high‐impedance probe (model HIP 511; Natus Neurology ‐ Grass, Warwick, RI, USA), amplified, and filtered (0.3–1 kHz; model P 511; Natus Neurology ‐ Grass). The compound action potentials were displayed on a computer monitor (Spike 2). The compound potentials from 15 stimulus presentations were summed, and the areas under A*δ* (i.e., group III) and the C (i.e., group IV) waves were integrated (Spike 2). Statistical analysis was performed with repeated measures ANOVA using GraphPad Prism (San Diego, CA); when appropriate Holm–Sidak post hoc tests were performed. The criterion for statistical significance was *P* < 0.05.

## Results

**Figure 1:**
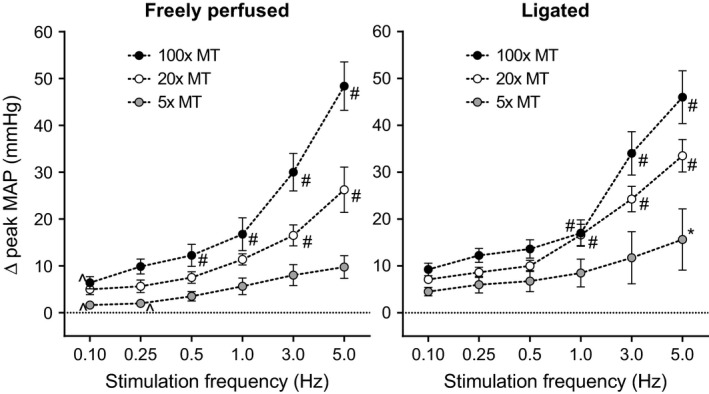
Ligated and freely perfused rats show similar reflexive blood pressure responses to direct afferent nerve stimulation. Averaged peak change in mean arterial pressure (mmHg) for freely perfused (left, *n *= 8/group) or ligated (right, *n *= 8/group) rats in response to direct afferent nerve stimulation at five times (gray circles), 20 times (white circles), or 100 times (black circles) motor twitch threshold (MT) across several stimulation frequencies. *Only one value, five times MT and 5 Hz, was statistically different (*P *= 0.047) between freely perfused and ligated rats. ^Only three values did not differ significantly from baseline. #Significantly different than stimulation at five times MT of the same frequency.

**Figure 2:**
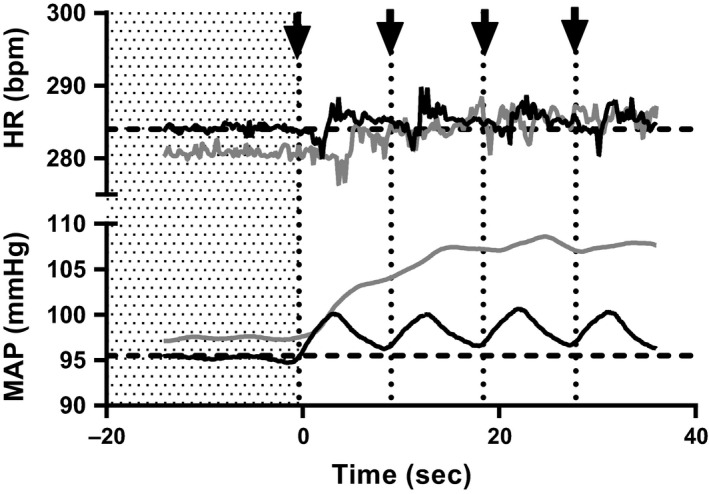
The reflexive blood pressure response recovers between each afferent nerve stimulation at 0.1 Hz frequency. Mean arterial pressure (MAP) and heart rate (HR) values recorded from a rat during tibial nerve stimulation at 100× motor twitch threshold and 0.1 Hz (black traces) and 0.25 Hz (gray traces). In all rats, both MAP and HR returned to near baseline levels (shaded area, dashed lines) between stimulations at 0.1 Hz (arrows, dotted lines), while stimulation at 0.25 Hz showed a more consistent increase.

Pressor responses to electrical stimulation of the tibial nerve in rats with freely perfused femoral arteries were compared to those in rats with ligated femoral arteries (eight freely perfused rats and eight ligated rats per stimulation intensity). Baseline mean arterial pressures were similar between freely perfused and ligated rats (Table 1). Regardless of the frequency or the current intensity used, we found little if any difference between freely perfused and ligated rats in the pressor responses to stimulation of the tibial nerve (Fig. 1). Most importantly, the threshold frequency needed to evoke and sustain a pressor response in both groups of rats was 0.25 Hz. Although we observed a significant blood pressure response at 0.1 Hz in certain groups, we found that the increase was transient and returned to baseline values between subsequent stimulations (Fig. 2). Increases in frequency evoked graded increases in arterial pressure in both groups of rats (Fig. 1). Similarly, increases in the current intensity of the pulses, which were expressed as multiples of motor threshold, evoked graded increases in arterial pressure at stimulation frequencies starting at 0.5 Hz and continuing to 5 Hz (*P* < 0.05; Fig. 1). Electrical stimulation of the tibial nerve at all frequencies and motor thresholds evoked trivial increases in heart rate in both freely perfused and ligated rats (Table 2).

**Table 1:** Baseline mean arterial pressures (mmHg, ±SEM) were similar between rats with freely perfused (FP) hind limbs and rats whose hind limbs were ligated for 72 h before stimulation at 5, 20, or 100 times the motor threshold current and at six different frequencies. There were no significant differences between baseline blood pressures in freely perfused rats and ligated rats

λ (Hz)	Baseline mean arterial pressures (mmHg)					
	5×		20×		100×	
	FP (*n* = 8)	Ligated (*n* = 8)	FP (*n* = 8)	Ligated (*n* = 8)	FP (*n* = 8)	Ligated (*n* = 8)
0.1	123 ± 11	101 ± 8	106 ± 10	106 ± 6	104 ± 9	100 ± 4
0.25	111 ± 10	108 ± 6	102 ± 9	105 ± 5	104 ± 10	106 ± 5
0.5	109 ± 11	104 ± 7	110 ± 8	109 ± 4	104 ± 9	98 ± 5
1.0	121 ± 9	103 ± 7	102 ± 8	108 ± 6	102 ± 9	97 ± 7
3.0	111 ± 10	107 ± 7	111 ± 9	108 ± 6	104 ± 10	102 ± 5
5.0	116 ± 12	103 ± 9	109 ± 10	104 ± 8	100 ± 10	104 ± 8

**Table 2:** Electrical stimulation of the tibial nerve at all frequencies and motor thresholds evoked trivial increases in heart rate (bpm, ±SEM) in both freely perfused (FP) and ligated rats. Mean baseline heart rate values for each group are displayed in parentheses

λ (Hz)	Mean heart rate increase during stimulation (bpm)					
	5×		20×		100×	
	FP (*n* = 8)	Ligated (*n* = 8)	FP (*n* = 8)	Ligated (*n* = 8)	FP (*n* = 8)	Ligated (*n* = 8)
0.1	1.0 ± 0.4 (301 ± 26)	1.0 ± 0.4 (337 ± 16)	2.0 ± 0.3 (313 ± 31)	3.1 ± 0.7 (359 ± 17)	2.9 ± 0.7 (381 ± 19)	1.3 ± 0.5 (357 ± 14)
0.25	1.4 ± 0.7 (297 ± 23)	2.0 ± 0.6 (335 ± 16)	2.3 ± 0.7 (315 ± 31)	3.3 ± 1.0 (362 ± 17)	4.5 ± 0.8 (380 ± 18)	3.0 ± 0.7 (356 ± 14)
0.5	1.8 ± 0.7 (299 ± 24)	2.3 ± 0.8 (335 ± 17)	2.0 ± 0.7 (315 ± 32)	6.4 ± 1.1 (364 ± 15)	7.0 ± 1.3 (380 ± 19)	4.9 ± 1.1 (351 ± 14)
1.0	2.9 ± 1.3 (290 ± 25)	1.9 ± 0.5 (340 ± 16)	4.6 ± 1.1 (311 ± 31)	11.0 ± 2.2 (362 ± 16)	7.1 ± 1.8 (382 ± 18)	7.8 ± 2.5 (356 ± 16)
3.0	2.1 ± 1.0 (300 ± 24)	3.6 ± 1.0 (337 ± 16)	7.8 ± 2.5 (310 ± 30)	13.6 ± 1.5 (366 ± 17)	11.4 ± 2.9 (384 ± 20)	10.4 ± 2.5 (355 ± 14)
5.0	3.0 ± 0.9 (299 ± 25)	5.1 ± 2.1 (334 ± 17)	5.8 ± 1.3 (318 ± 31)	16.4 ± 2.8 (364 ± 16)	12.6 ± 3.6 (382 ± 21)	12.4 ± 3.4 (351 ± 14)

**Figure 3:**
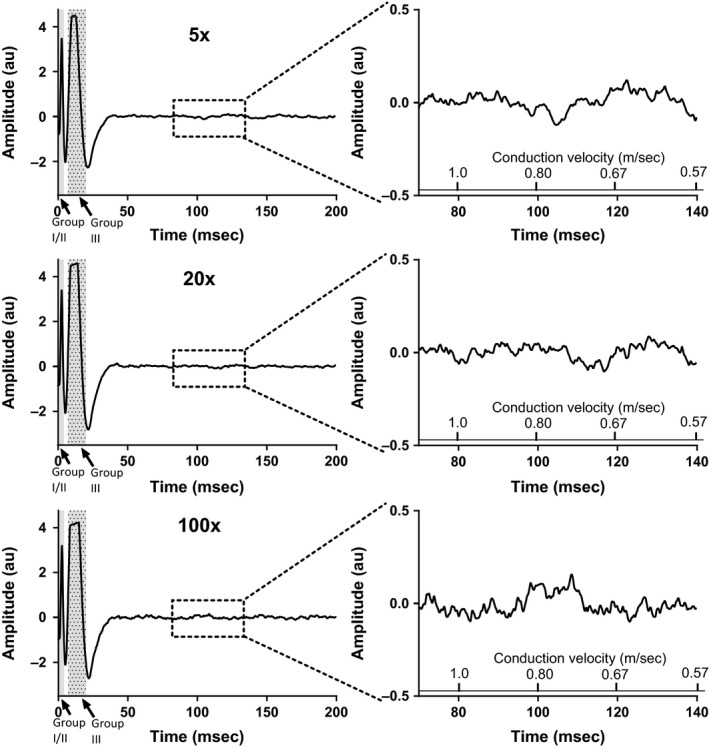
Tibial nerve stimulation at 100× motor twitch threshold evokes compound action potentials indicative of group I, II, III, and IV afferents. Left: representative compound action potential recorded from an L5 dorsal root in response to stimulation (0.01 msec duration) of the tibial nerve at five times (upper), 20 times (middle), and 100 times (lower) motor twitch threshold. Traces shown are the average of 15 sweeps. Right: enlargement of dotted box region on left showing time period during which a group IV afferent wave would be expected, corresponding to a conduction velocity of 0.57–1.15 m/sec.

**Figure 4:**
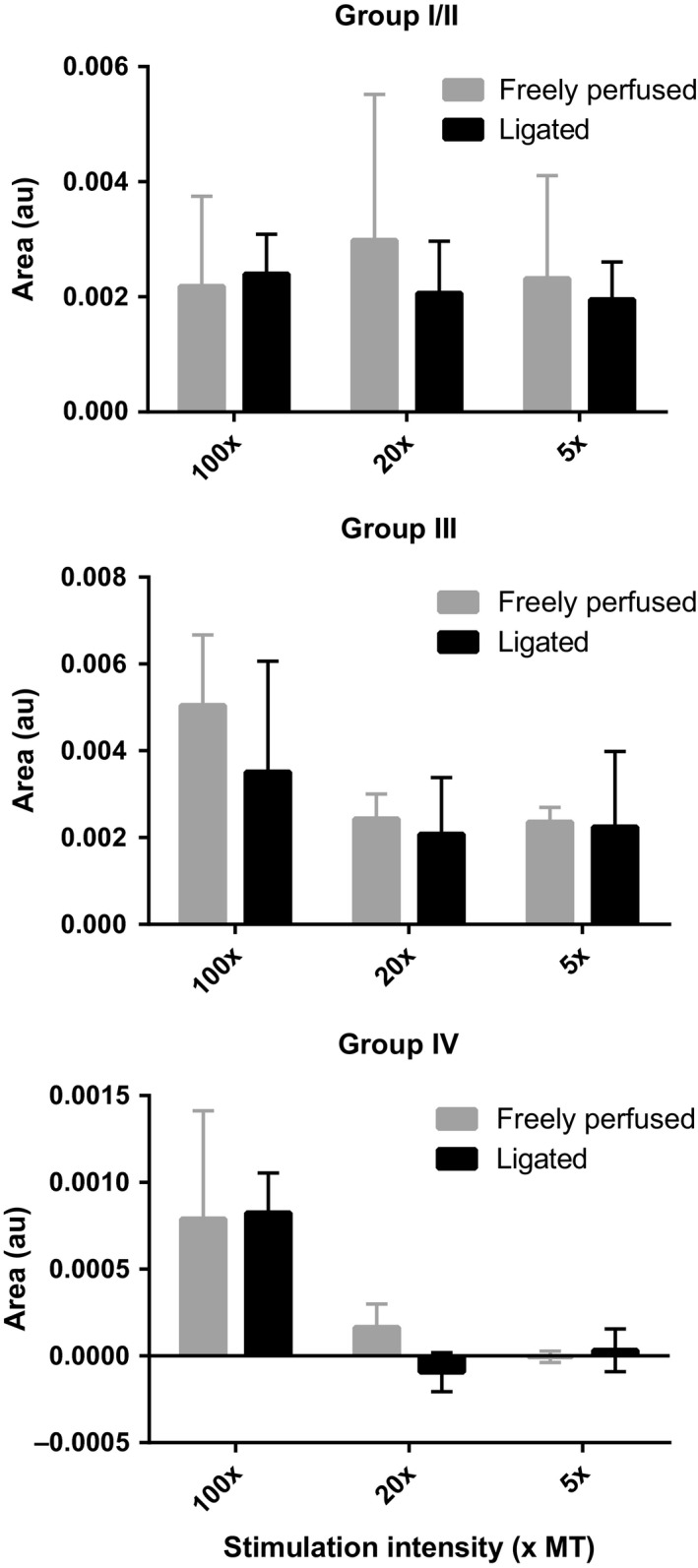
Integrated areas of compound action potentials were similar between freely perfused and ligated rats. Curve areas for group I/II (top), group III (middle) and group IV (bottom) compound action potential waves, as represented in Figure 3. No significant differences were observed between freely perfused (gray bars) and ligated (black bars) rats. Group IV areas for 5× and 20× stimulation intensities were calculated across the same region as the 100× wave, as group IV waves were not observed at the lower stimulation intensities.

Next, the compound action potential was recorded from either the L4 or L5 dorsal root that was evoked by single‐pulse stimulation of the tibial nerve at three current intensities, namely 5, 20, and 100 times the motor threshold. In three freely perfused and three ligated rats, we found that stimulating the tibial nerve at five times the motor threshold evoked compound action potentials indicative of group I, II, and III afferent activation. Likewise, in these six rats, stimulating the tibial nerve at 20 times the motor threshold evoked compound action potentials indicative of group I, II, and III activation. In contrast, stimulation at 100 times the motor threshold evoked a compound action potential indicative of group IV afferent activation in addition to waves indicative of groups I, II, and III activation (Fig. 3). There were no differences between the integrated areas of any of the compound action potentials evoked by tibial nerve stimulation in freely perfused rats and those evoked by stimulation in ligated rats (Fig. 4).

## Discussion

The sensory arm of the exercise pressor reflex arc is comprised of thinly myelinated group III afferents and unmyelinated group IV afferents (Coote and Perez‐Gonzalez 1970; McCloskey and Mitchell 1972). The endings of these afferents are located in the walls of small vessels and connective tissue within the muscles (von During and Andres 1990). Group Ia, Ib, and group II muscle afferents (i.e., spindles and Golgi tendon organs) do not contribute to the afferent arm of the exercise pressor reflex (Hodgson and Matthews 1968; McCloskey and Mitchell 1972; McCloskey et al. 1972; Waldrop et al. 1984). Smith et al. (2001) showed that static contraction of the hind limb muscles of decerebrated rats with freely perfused femoral arteries reflexively increased arterial pressure and heart rate, findings that were independently replicated by Tsuchimochi et al. (2010).

Ligating a femoral artery for 72 h before the start of the experiment was shown to substantially increase the magnitude of the exercise pressor reflex over that seen within the same rats having freely perfused femoral arteries on the opposite side (Tsuchimochi et al. 2010). In subsequent experiments, Stone et al. (2015) found that static contraction of the triceps surae muscles increased group III and IV afferent activity by about 0.5 impulses per second in rats with freely perfused femoral arteries and by about 1.0 impulses per second in rats with ligated femoral arteries. Although these electrophysiological findings were consistent with reflex findings reported by Tsuchimochi et al. (2010), the question arose whether the relatively small increases in group III and IV muscle afferent activity could account for the reflex increases in arterial pressure evoked by static contraction in rats with either freely perfused or ligated femoral arteries. This question gained importance when viewed in light of similar experiments in dogs in which reflex pressor responses were evoked by stimulating hind limb muscle nerves at frequencies of 20–100 Hz (Mitchell et al. 1968; Rybicki and Kaufman 1985). In these experiments, frequencies below 20 Hz were not used in combination with current intensities sufficient to activate unmyelinated muscle afferents (Mitchell et al. 1968; Rybicki and Kaufman 1985).

In this study, we found that the threshold frequency required to evoke a reflex pressor response when electrically stimulating the tibial nerve was 0.25 Hz in both rats with freely perfused femoral arteries and in rats with ligated femoral arteries. We note with interest that this frequency is the same as that reported by Sato et al. (1981) to reflexively increase heart rate in vagotomized, paralyzed cats anesthetized with chloralose‐urethane. We also found that when the current intensity applied to the tibial nerve recruited both group III and IV afferents (i.e., 100 times the motor threshold), the pressor responses approached those evoked by static contraction in rats with freely perfused femoral arteries. This was the case regardless of whether we were stimulating a rat with a freely perfused or ligated femoral artery. Our interpretation of this finding is that the greater pressor response to contraction in rats with ligated, compared to freely perfused femoral arteries was caused by differences in the contraction‐induced responses of group III and IV muscle afferents (Stone et al. 2015); and was not caused by differences in the central processing of afferent input caused by ligation. Correspondingly, if femoral arterial ligation had altered the central processing of group III and IV afferent input to the dorsal horn of the spinal cord, then the pressor response to electrical stimulation of the tibial nerve should have evoked a greater pressor response in the rats whose femoral arteries were ligated than in those that were freely perfused.

The interpretation of data obtained by electrically stimulating the tibial nerve has two important limitations. First, stimulation may have activated afferent fibers that innervated skin and joints as well as afferent fibers that innervated the triceps surae muscles. Some cutaneous afferents, when stimulated electrically, can reflexively decrease arterial pressure (Coote and Perez‐Gonzalez 1970) and therefore may have opposed the pressor effects seen in our experiments. We attempted to minimize the recruitment of cutaneous and joint afferents by placing the electrode as close as possible to the triceps surae muscles. Second, electrical stimulation at any of the frequencies used in our experiments evoked afferent impulse activity with regular interspike intervals, whereas natural stimulation, such as that evoked by muscle contraction, evoked afferent impulse activity with irregular interspike intervals (Kaufman et al. 1983; Mense and Stahnke 1983; Stone et al. 2015). Irregular interspike intervals may result in temporal summation that would amplify postsynaptic EPSPs in the dorsal horn, thereby exaggerating the pressor response to contraction.

In our experiments, we found that electrical stimulation of group III and IV afferents in the tibial nerve increased heart rate, but the effect was small and was not graded as it was for arterial pressure. Two factors are probably responsible for this finding. First, we paralyzed the rats with pancuronium, an agent which increased baseline heart rate by its vagolytic action (Marshall 1973; Gursoy et al. 2011). Our use of pancuronium might have limited stimulation‐induced increases in cardiac output, which in turn might have contributed to an increase in arterial pressure. Paralyzation was necessary in order to prevent contraction from stimulating the afferents. Second, the arterial baroreceptors were intact in our experiments; they most likely buffered any cardioaccelerator effects arising from the stimulation of hind limb afferents.

## Conclusions

In conclusion, we have shown that electrically discharging group III and IV hind limb somatic afferents at low frequencies similar to those evoked by static contraction reflexively increased mean arterial pressure. We suggest that most of the group III and IV afferents stimulated in our experiments innervated the triceps surae muscles, although we cannot completely exclude the possibility that afferents innervating other structures were also stimulated. We also found that the reflex pressor responses to electrical stimulation of hind limb afferents were not dependent on whether the femoral artery was freely perfused or was ligated. This finding suggests that spinal and medullary processing of group III and IV afferent input plays no role in augmenting the pressor response to contraction caused by ligating the femoral artery in decerebrate rats. Consequently, efforts toward ameliorating the deleterious effects of the exaggerated exercise pressor reflex and accompanying claudication seen in human peripheral artery disease should prioritize the primary muscle afferents, and not central neural processing of afferent input.

## Conflict of Interest

None declared.
